# The Culture Environment Influences Both Gene Regulation and Phenotypic Heterogeneity in *Escherichia coli*

**DOI:** 10.3389/fmicb.2018.01739

**Published:** 2018-08-15

**Authors:** Ashley Smith, Agnieszka Kaczmar, Rosemary A. Bamford, Christopher Smith, Simona Frustaci, Andrea Kovacs-Simon, Paul O’Neill, Karen Moore, Konrad Paszkiewicz, Richard W. Titball, Stefano Pagliara

**Affiliations:** ^1^Living Systems Institute, University of Exeter, Exeter, United Kingdom; ^2^Biosciences, University of Exeter, Exeter, United Kingdom

**Keywords:** phenotypic heterogeneity, *Escherichia coli*, persisters, metabolism, bacterial physiology, antibiotics, gene-expression profiling, KEGG pathways

## Abstract

Microorganisms shape the composition of the medium they are growing in, which in turn has profound consequences on the reprogramming of the population gene-expression profile. In this paper, we investigate the progressive changes in pH and sugar availability in the medium of a growing *Escherichia coli* (*E. coli*) culture. We show how these changes have an effect on both the cellular heterogeneity within the microbial community and the gene-expression profile of the microbial population. We measure the changes in gene-expression as *E. coli* moves from lag, to exponential, and finally into stationary phase. We found that pathways linked to the changes in the medium composition such as ribosomal, tricarboxylic acid cycle (TCA), transport, and metabolism pathways are strongly regulated during the different growth phases. In order to quantify the corresponding temporal changes in the population heterogeneity, we measure the fraction of *E. coli* persisters surviving different antibiotic treatments during the various phases of growth. We show that the composition of the medium in which β-lactams or quinolones, but not aminoglycosides, are dissolved strongly affects the measured phenotypic heterogeneity within the culture. Our findings contribute to a better understanding on how the composition of the culture medium influences both the reprogramming in the population gene-expression and the emergence of phenotypic variants.

## Introduction

Within isogenic populations there may be substantial cell-to-cell heterogeneity in terms of metabolic activity (Nikolic et al., 2013; Şimşek and Kim, 2018), growth rate (Kotte et al., 2014), substrate assimilation (Sheik et al., 2016), compound secretion (Veening et al., 2008), virulence (Arnoldini et al., 2014), and resistance to stress (Balaban et al., 2004). This heterogeneity has been observed across all the domains of life and arises from the inherent random nature of biochemical reactions (Elowitz et al., 2002; Kaern et al., 2005; Lidstrom and Konopka, 2010). Phenotypic heterogeneity may allow some individual cells to survive shifts in the environmental conditions, and thus permitting the population to withstand fluctuating environments (Balaban et al., 2004; Ackermann, 2015; Venturelli et al., 2015; Schreiber et al., 2016; Bódi et al., 2017). It has also been suggested that phenotypic heterogeneity can accelerate evolutionary adaptation to different environmental challenges (Beaumont et al., 2009; New et al., 2014). The culture environment in turn affects the population transcriptome. For instance, pH has been shown to regulate genes involved in catabolism and transport (Hayes et al., 2006), whereas glucose-lactose diauxie induces the downregulation of amino acid biosynthesis and aerobic metabolism genes (Chang et al., 2002). Additionally, changes in gene-expression levels in response to nutritional changes are strongly linked to growth rate and cell size (Weart et al., 2007; Scott et al., 2010; Chien et al., 2012; Yao et al., 2012). Moreover, it has been suggested that a reduction in cell size increases the heterogeneity in gene-expression within the population (Kaern et al., 2005).

However, only a small subpopulation of bacteria shows observable physiological variations, such as growth rate that is more than twofold different than the remainder of the population (Lidstrom and Konopka, 2010). Therefore, the identification and study of such small subpopulations can be challenging but can be simplified by analyzing the functional consequences of a given case of phenotypic heterogeneity (Ackermann, 2015).

For example, persister cells are a small proportion of a clonal microbial population that can survive otherwise lethal doses of antibiotics and resume growth shortly after removing the antibiotic (Hansen et al., 2008; Lewis, 2010; Maisonneuve et al., 2013), but without acquiring genetic changes that confer antibiotic resistance. In this paper we used persister cell formation as a proxy for phenotypic heterogeneity. Persister cells have been observed across all the domains of life (Lewis, 2010; Hangauer et al., 2017; Megaw and Gilmore, 2017) and are believed to contribute to the survival of bacteria in biofilms exposed to antibiotics (LaFleur et al., 2006; Lewis, 2010) and to chronic infections in immunosuppressed hosts (Mulcahy et al., 2010; Maisonneuve and Gerdes, 2014).

Persisters can form stochastically as a result of fluctuations in gene-expression (Amato et al., 2013). However, a variety of environmental factors favor persister formation, including subinhibitory concentrations of antibiotics (Amato et al., 2013), nutrient limitation (Vega et al., 2012), intra-species interactions (Bernier et al., 2013), starvation (Fung et al., 2010), and in the case of pathogens, interactions with the host (Helaine et al., 2014). Amato et al. (2013) showed that diauxic growth contributes to persister cell formation, whereas another study by the same group showed that nutrient transitions contributed to persister formation within bacterial biofilms (Amato and Brynildsen, 2014). Keren et al. reported that the number of ampicillin or ofloxacin persisters increased from lag to stationary phase (Keren et al., 2004a). However, the temporal windows when there are substantial increases in the formation of persisters to different antibiotics during growth of *Escherichia coli* (*E. coli*) on lysogeny broth (LB) have yet to be defined. Moreover, gene-expression profiling has been carried out on both exponential and stationary phase *E. coli* O157 growing on 3-(*N*-morpholino)propanesulfonic acid (MOPS) minimal medium supplemented with 0.1% glucose (Bergholz et al., 2007). However, the changes in the transcriptome throughout the growth cycle of *E. coli* K12 growing in LB remain to be determined, despite this being an experimental model system employed in microbiology, biotechnology, and molecular biology.

In this paper, we report the changes in sugar levels and pH and the associated reprogramming in gene-expression during the transitions between the different phases of *E. coli* growth. We then investigate the phenotypic heterogeneity within the *E. coli* population throughout the growth cycle by using persister formation, in response to ampicillin, gentamicin, or ofloxacin as a proxy for studying cellular heterogeneity. Our findings will be instrumental for investigations into the mechanisms underlying microbial survival in transitioning environments and provide key transcriptomic data for a commonly used model in many bacterial studies.

## Materials and Methods

### Chemicals and Culture Preparation

All chemicals were purchased from Fisher Scientific or Sigma-Aldrich unless otherwise stated. LB medium (10 g/L tryptone, 5 g/L yeast extract, and 10 g/L NaCl, Melford) and LB agar plates (LB with 15 g/L agar) were used for planktonic growth and enumeration of colony-forming units (CFUs), respectively. *E. coli* BW25113 was purchased from Dharmacon (GE Healthcare). A single colony of *E. coli* BW25113 was grown in 200 ml fresh LB in a shaking incubator at 200 rpm and 37°C for 17 h (**Supplementary Figure S1A**). After 17 h incubation, the culture was diluted 1:1000 in fresh LB and growth was measured hourly by taking three aliquots that were then centrifuged (13,000 *g* for 5 min), the supernatant was removed, the pellet was resuspended in phosphate-buffered saline (PBS), and serial dilutions were plated on LB agar for CFU counts (**Supplementary Figures S1B,C,H**). This experiment allowed us to determine that the culture was in stationary phase at *t* = 17 h (left axis in **Supplementary Figure S2**). In order to avoid introducing any bias in our measurements (Luidalepp et al., 2011), we used the same LB autoclaving conditions in all our assays. The relatively small error bars in our measurements and in other recent reports (Orman and Brynildsen, 2016; Radzikowski et al., 2016) demonstrate the suitability of autoclaved LB for these microbiological assays.

### Characterizing the Bacterial Environment

A culture was prepared as described above and eighty-one 100 μl aliquots were added to individual wells of a 96-well plate (three technical replicates in biological triplicates for each of the nine time points were investigated). The remaining wells were filled with fresh LB for blank measurements. The plate was placed in a preheated (37°C) Infinite^®^ 200 PRO plate reader (TECAN) shaking at 200 rpm. To quantify bacterial growth in this assay, optical density at 595 nm (OD_595_) was measured hourly in nine selected wells for each time point. Bacterial growth measured via the plate reader method (right axis in **Supplementary Figure S2**) was comparable to that measured via CFU counts in cultures growing in 200 ml flasks (left axis in **Supplementary Figure S2**). To quantify the amount of reducing sugars, preheated (100°C) Benedict’s reagent (Sigma-Aldrich) was then added to the same wells according to the manufacturer’s instructions and absorbance at 490 nm was measured after 15 min incubation. The absolute sugar concentration was determined by extrapolation through a standard curve of known glucose concentration (**Supplementary Figure S3**). This was obtained by adding glucose in MilliQ water at concentrations of 125, 250, 500, or 1000 μM in triplicate in a 96-well plate. Preheated (100°C) Benedict’s reagent was then added to the same wells and the absorbance at 490 nm was measured after a 15 min incubation. The average reading from three wells containing only MilliQ water was subtracted from the readings of the glucose containing wells. These blank subtracted readings are reported in **Supplementary Figure S3** together with a linear regression fitting of the experimental data. In order to measure the culture pH, the probe of a PH-100 ATC pH meter (with an accuracy of pH 0.01, Voltcraft) was immersed in a separate culture prepared as described above and the pH was recorded hourly. The measurements were taken in at least three biological replicates.

### Transcriptomic and qPCR Analysis

A culture was prepared as described above. Immediately after dilution (0 h), 500 μl aliquots were taken from the overnight (17 h) culture and 1, 2, 3, 3.5, 4, 4.5, 5, 6, or 7 h after dilution in fresh LB (1:1000) and were incubated at 200 rpm and 37°C as described above. The RNA of the cells contained in each aliquot was stabilized using RNAprotect Bacteria Reagent (Qiagen). Extraction was performed with RNeasy Mini Kit (Qiagen) and DNA removal with DNase I (RNase-free, Ambion), using the recommended protocols. RNA concentration and purity were determined using a 2100 Bioanalyzer (Agilent). cDNA libraries from all samples with an RNA integrity number (RIN) greater than eight were prepared and then sequenced using Illumina HiSeq 2500. The paired reads were trimmed and sequencing adaptors were removed using fastq-mcf. RNA ERCC spike-in control sequences were removed using bowtie version 1.0.0, and the remaining reads were aligned to the reference genome using tophat2 version 2.1.0. The gene-expression was quantified using HTseq-count. DESeq2 v1.6.3 was used to normalize the raw transcript reads for all genes by using the median-ratio normalization method and for library size (Love et al., 2014). To reduce the number of false-positive results, the log_2_ fold changes were shrunk toward zero for lowly expressed genes and the adjusted *p*-values were calculated using a false discovery rate (FDR) of 0.1. We then determined the log_2_ fold change in the normalized transcript reads for each gene at different time points, relative to the normalized transcript reads in the overnight stationary phase sample (*t* = 17 h). In order to identify the variables that best differentiate the data, as well as to determine how well-clustered the replicates were, we performed principal component analysis (PCA) using DESeq2 and a built-in R method (prcomp) on the top 500 expressed genes. These genes were normalized using a regularized log transform prior to PCA to allow better visualization of the trends and clusters that may otherwise remain hidden. The data shown represent the first (PC1) and second principal components (PC2). The clustering of the time point replicates indicates a high level of reproducibility in our data. During the three different growth phases the top 10% of upregulated and downregulated genes, based on their log_2_ fold change, were identified and goseq was used to identify overrepresented pathways in the Kyoto Encyclopedia of Genes and Genomes (KEGG) (Ogata et al., 1999; Kanehisa et al., 2016, 2017). In order to check the results, qPCR was performed on the same aliquots on a StepOnePlus^TM^ Real-Time PCR System for selected genes. Both RNA-seq and qPCR measurements were performed in biological triplicates.

### MIC Determination

The minimum inhibitory concentration (MIC) of the employed antibiotics against *E. coli* BW25113 was determined using a 96-well plate method. *E. coli* was grown for 17 h in LB containing different concentrations of ampicillin (0.5–512 μg ml^-1^), ofloxacin (0.0625–64 μg ml^-1^), or gentamicin (0.125–128 μg ml^-1^) and the OD_595_ was measured hourly. The MICs were measured as the lowest concentrations at which the OD_595_ was the same as the control (bacteria-free LB) and were determined as 5, 4, and 0.125 μg ml^-1^ for ampicillin, gentamicin, and ofloxacin, respectively.

### Persister Enumeration

A culture was prepared as described above and during mid-exponential phase (*t* = 3 h after dilution) the respective antibiotics were added to the culture to reach a concentration of 25 × MIC, with persister levels typically not varying above this concentration of antibiotics (Johnson and Levin, 2013). Every 30 min an aliquot was taken from the treated culture, centrifuged (13,000 *g* for 5 min), re-suspended in PBS, and plated on LB agar plates. The plates were incubated and CFUs were determined the following day. For each antibiotic, the fraction of persister cells plateaued after 3 h of treatment, as previously reported (Johnson and Levin, 2013), confirming that we were studying persister subpopulations rather than antibiotic-tolerant populations (Brauner et al., 2016).

In order to enumerate persisters based on the effect of different antibiotics during the various phases of growth, a culture was prepared as described above (**Supplementary Figures S1A,B**). Nine 500 μl aliquots were withdrawn from the growing culture hourly (**Supplementary Figures S1C,E**). Three of them were used for untreated controls, the aliquots were centrifuged (13,000 *g* for 5 min), supernatant was removed, the pellet was resuspended in PBS, and serial dilutions were plated on LB agar (**Supplementary Figure S1H**). Three aliquots were supplied with 500 μl LB (1:1 dilution) containing 50 × MIC of one of the three above specified antibiotics (final concentration 25 × MIC) and were returned to the shaking incubator (**Supplementary Figure S1F**). After 3 h, these aliquots were centrifuged, the supernatant was removed, and the pellet was re-suspended in PBS. Serial dilutions were then performed and plated on LB agar (**Supplementary Figure S1I**). Three aliquots were injected with 10 μl of one of the three above specified antibiotics to reach a final concentration of 25 × MIC and returned to the shaking incubator (**Supplementary Figure S1G**). After 3 h these aliquots were centrifuged, the supernatant was removed, the pellet was re-suspended in PBS, serially diluted and plated on LB agar (**Supplementary Figure S1J**).

## Results

### Nutritional and Chemical Environment of a Growing *E. coli* Culture

We investigated how the sugar content and the pH of the growth medium changed over time. Notably, both quantities are known to affect the outcome of antibiotic treatment (Allison et al., 2011; Cama et al., 2014). The measured concentration of fermentable sugars in the LB medium we employed was 163 ± 35 μM. A previous study found that LB contained less than 100 μM fermentable sugars by using a genetic approach based on a *hemA* deletion mutant unable to grow in the absence of fermentable sugars (Sezonov et al., 2007). This discrepancy could be due to the different sources of LB and the different techniques used to quantify the sugar concentrations. This further emphasizes the added value of carrying out the simple assay described in Section 3.2 to quantify the concentration of fermentable sugars during bacterial growth.

**FIGURE 1:**
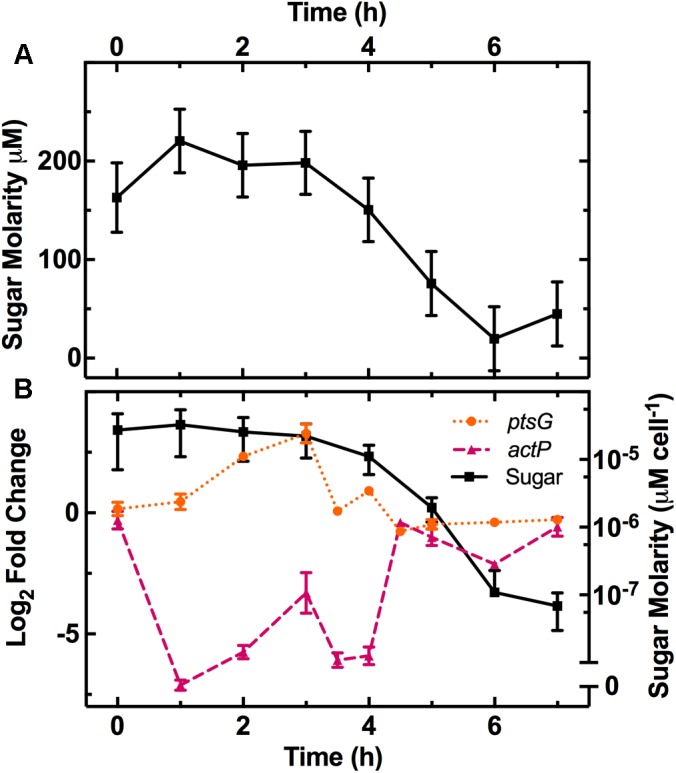
Characterization of the physical changes in the bacterial environment. **(A)** Dependence of the concentration of reducing sugars in the culture on the time elapsed from dilution in LB medium of an overnight *E. coli* culture. **(B)** Dependence of sugar concentration per cell (squares, right axis) and the expression of the glucose (*ptsG*, circles, left axis) and acetate (*actP*, triangles, left axis) related genes on time elapsed from dilution in LB medium. Gene-expression is reported as the log_2_ fold change with respect to the measurements on the overnight samples (*t* = 17 h). Data and error bars are the mean and standard error of the mean (SEM) calculated on measurements obtained in biological triplicate.

After *E. coli* inoculation into LB medium, we measured the remaining sugar concentration at various intervals throughout the growth cycle (**Figure 1A**). We calculated the corresponding concentration of sugar available per bacterium (squares in **Figure 1B**) by dividing the measured sugar concentration by the measured number of bacteria in the culture (full symbols in **Supplementary Figure S2**, left axis). This revealed a one order of magnitude decrease in the sugar available per bacterium between 3 and 6 h after inoculation, when the culture transitioned from exponential to stationary growth-phase.

We also measured the pH of the culture throughout the growth cycle (**Supplementary Figure S4**). The pH decreased from 6.8 and reached it’s most acidic value of 6.2 during the exponential phase at *t* = 4 h, then rose up to a maximum of 7.0 during the stationary phase at *t* = 7 h. We explain this finding by considering that the culture environment is acidified by the excretion of acetate during aerobic fermentation, resulting from bacterial growth on carbohydrates during exponential phase (Kleman and Strohl, 1994). However, upon exhaustion of these carbohydrates, the bacteria use alternative carbon sources such as amino acids and other gluconeogenic substrates (Sezonov et al., 2007), resulting in the production and excretion of ammonia that increases the culture pH. Losen et al. (2004) did not observe the same growth-phase dependence for the pH of a growing *E. coli* culture. However, their assay was performed using a different *E. coli* strain (ATCC 53323) and different culture conditions including a different LB supplier, a one order of magnitude smaller LB volume and a one order of magnitude higher inoculum concentration. All together, our data complement our existing knowledge on the changes occurring in the medium composition during *E. coli* growth in LB (Losen et al., 2004; Sezonov et al., 2007).

### Changes in Gene-Expression During the Growth Cycle

The gene-expression profile of bacterial populations is profoundly affected by changes in the culture (Hua et al., 2004; Bergholz et al., 2007; Klumpp and Hwa, 2015; Vital et al., 2015). However, to the best of our knowledge, this is the first study reporting the progressive reprogramming of the gene-expression profile of *E. coli* growing in LB throughout the different phases of growth.

In order to study the effect of the changing nutritional or chemical environment of the culture on the population transcriptome, we measured gene-expression profile in aliquots taken at different stages of growth in biological triplicates. **Supplementary Table S1** reports, for each gene, the mean and SEM of the normalized transcript reads measured in the samples taken at *t* = 17 h post inoculation. **Supplementary Table S1** also reports the mean and SEM of the log_2_ fold change in normalized transcript reads in the samples taken at *t* = 0, 1, 2, 3, 3.5, 4, 4.5, 5, 6, or 7 h post inoculation relative to the *t* = 17 h sample. The mean relative error, averaged on the relative errors for the transcript reads of all genes at *t* = 17 h, is 24%, thus confirming good reproducibility across biological replicates. Indeed, this corresponds to a log_2_ fold change of 0.31, whereas in comparison, the average absolute log_2_ fold change in gene-expression at *t* = 2 h relative to *t* = 17 h is 1.9. We further confirmed the changes in gene-expression of selected genes using qPCR (**Supplementary Figure S5**).

**FIGURE 2:**
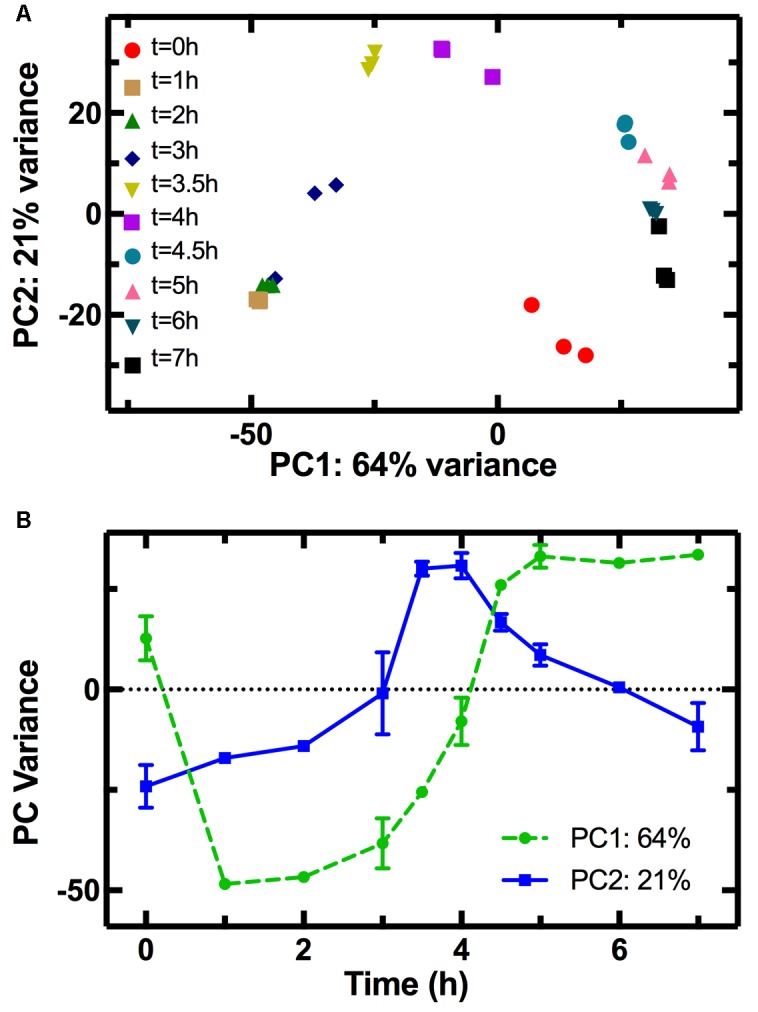
Principal component analysis (PCA) of the transcriptome at different stages of growth. **(A)** Correlation between the first (PC1) and second (PC2) principal components of the transcriptome of samples taken in biological triplicate at different time points during *E. coli* growth in LB. The clustering of transcriptomes from the same time points confirms the reproducibility of our measurements. Furthermore, adjacent time points clustered close to each other forming a nearly complete cycle, the transcriptome measured at *t* = 0 being close to that measured at *t* = 7 h. **(B)** The temporal dependence of PC1 (circles) resembles that of *E. coli* growth (**Figure 3A**) suggesting that genes used for PC1 may play a role in adaptation to the exhaustion of nutrients, whereas the temporal dependence of PC2 (squares) resembles that of the culture pH (**Supplementary Figure S4**) suggesting that these genes may be involved in the adaptation to pH changes.

The PCA allowed clustering of the transcriptome profiles measured from the different biological replicates at each time point (**Figure 2A**), demonstrating good reproducibility of our cultures grown in shake flasks without the need for fermenter cultivation (Chang et al., 2002). The transcriptomes from adjacent time points clustered close to each other forming a nearly complete cycle, with the transcriptome measured at *t* = 0 being close to that measured at *t* = 7 h. Similarly, a cyclic transcriptional response of *E. coli* to acid adaptation was previously reported (Stincone et al., 2011). Furthermore, the transcriptomes measured at *t* = 0 and *t* = 1 h are simultaneously similar in terms of PC2 but different in terms of PC1 (**Figure 2B**), suggesting that part of the transcriptome rapidly adapts to changes in the nutritional environment. The population transcriptome then becomes increasingly different in PC1 (circles in **Figure 2B**). This suggests that the regulation of the genes used for PC1 analysis allows the culture to progressively adapt to an environment unfavorable for growth, as explained in the discussion below. On the other hand, the PC2 variance reveals that the transcriptomes at *t* = 3.5 and *t* = 4 h differ the most from the transcriptome at *t* = 0 h. The PC2 variance for the *t* = 0 h transcriptome is instead similar to that of the *t* = 7 h transcriptome, a trend similar to the temporal dependence of the average division rate (**Supplementary Figure S6**) and a mirror image of the trend in pH (**Supplementary Figure S4**). This suggests that the regulation of the genes used in the PC2 analysis governs the cell division and metabolism machineries, which in turn drive the changes in the environmental pH. This is, to the best of our knowledge, the first time PCA is carried out on the transcriptome of an *E. coli* culture throughout its growth cycle.

The decrease in sugar levels in the medium parallels the regulation of a set of genes including *ptsG*, a glucose-specific phosphotransferase (Luli and Strohl, 1990), and the dedicated acetate uptake system *actP* (Luli and Strohl, 1990; **Figure 1B**). Expression of *ptsG* increases during the lag phase (circles in **Figure 1B**) when fresh medium is added to the culture and then decreases as the sugar concentration per bacterium decreases after *t* = 3 h (squares in **Figure 1B**). Bergholz et al. did not investigate gene-expression profile during lag phase but reported a similar downregulation of *ptsG* with a -3 log_2_ fold change between 4.5 and 5 h growth. In comparison, *actP* expression rapidly decreases between *t* = 0 h to *t* = 1 h as fresh medium is added to the culture before increasing at *t* = 4 h as sugars are metabolized and acetate becomes available in the environment (triangles in **Figure 1B**) as previously reported (Bergholz et al., 2007).

**FIGURE 3:**
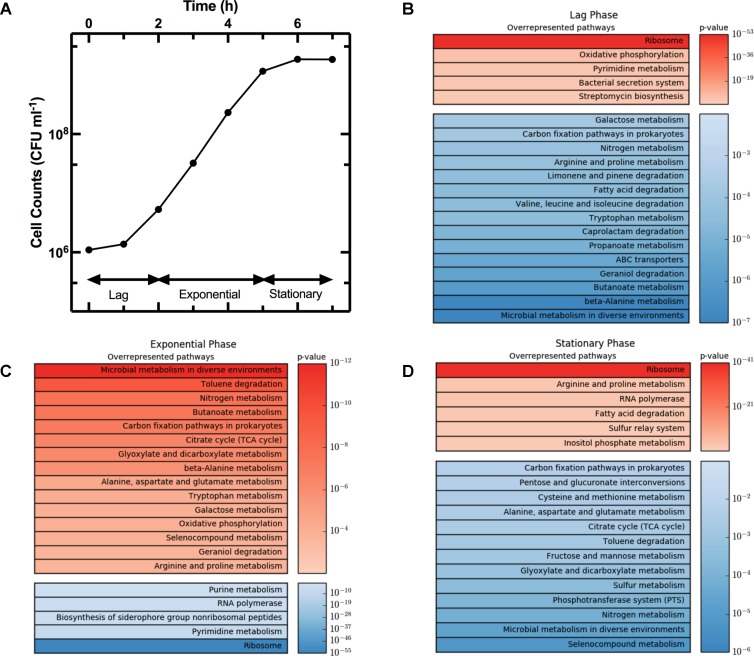
Regulation of KEGG pathways during different phases of the growth cycle. **(A)** Growth phases of an *E. coli* culture. **(B–D)** Heat map tables of the top twenty overrepresented KEGG pathways during lag, exponential, and stationary phase, respectively. Each top table reports upregulated genes (in red) while each bottom table reports downregulated pathways (in blue). Pathways were ranked by significance of their *p*-values. Both growth and gene-expression profile measurements were performed in biological triplicate. In **(A)** data and error bars are the mean and SEM of measurements. Error bars are small compared to the corresponding mean values and are hidden behind some of the data points.

The growth curve in **Figure 3A** shows the three characteristic phases of growth: lag phase between *t* = 0 h and *t* = 2 h, exponential phase from *t* = 2 h to *t* = 5 h, and stationary phase from *t* = 5 h onward. We considered gene regulation during each of these phases based on the log_2_ fold change in transcript levels at *t* = 2 h relative to *t* = 0 h, *t* = 5 h relative to *t* = 2 h, and *t* = 7 h relative to *t* = 5 h, respectively. Furthermore, for each growth phase we grouped the top 10% of upregulated genes, from the 4313 genes analyzed. Then for each KEGG pathway we determined the number of genes that were in the top 10% group. We then used goseq to calculate the probability of this number occurring when compared to the total number of genes in the pathway (*p*-value in **Figure 3**). For example, the KEGG pathway “Microbial metabolism in diverse environments” has 201 associated genes. Therefore, in the top 10% group of the upregulated genes from all pathways one would expect to find 20 genes associated to this KEGG pathway. However, in the top 10% group of upregulated genes during exponential phase, we identified 54 genes from the “Microbial metabolism in diverse environments” pathway. Therefore, this pathway was overrepresented in the 10% group of upregulated genes during exponential phase with a *p*-value of 1.12 × 10^-12^. We repeated this process for the top 10% downregulated genes, before ranking all the KEGG pathways by *p*-value, and reported the top 20 overrepresented pathways for the up- and downregulated genes (in red and blue, respectively, in **Figure 3**) during lag phase (**Figure 3B**), exponential phase (**Figure 3C**), and stationary phase (**Figure 3D**). **Supplementary Table S2** reports the *p*-value and number of genes in each of these pathways for lag, exponential, and stationary phase. We could not directly compare our results with previously reported datasets (Weber et al., 2005; Bergholz et al., 2007) because these studies did not employ the KEGG database. Therefore, we report below the expression of strongly regulated genes for each growth phase and discuss our findings in the context of data reported in previous studies investigating either persisters or the influence of the medium composition on gene regulation.

**FIGURE 4:**
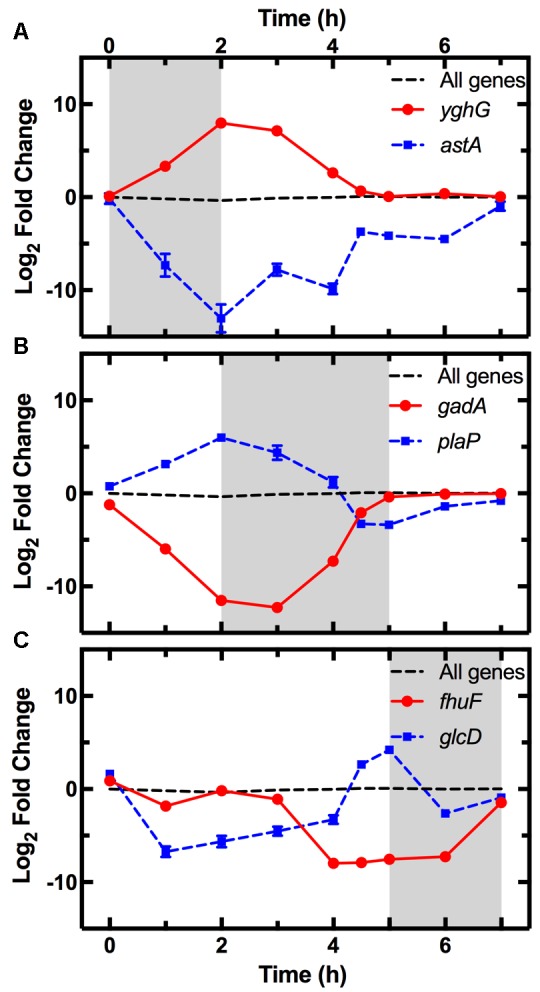
Pattern of expression of the top most upregulated and downregulated genes during each phase. Expression profiles of the most upregulated (circles) and downregulated (squares) genes between *t* = 0–2 h **(A)**, *t* = 2–5 h **(B)**, and *t* = 5–7 h **(C)** from dilution in LB medium of an overnight *E. coli* culture. Gene expression is reported as the log_2_ fold change with respect to the measurements on the overnight samples (*t* = 17 h). The average expression dynamics for the whole transcriptome (dashed line) remains relatively constant throughout the different phases of growth. The expression dynamics for all the 4373 analyzed genes is reported in **Supplementary Table S1**. Data and error bars are the mean and SEM of measurements obtained on biological triplicates. Error bars are hidden behind some of the data points.

The average expression dynamics for the whole transcriptome (dashed line in **Figure 4**) remained relatively constant throughout the different phases of growth. However, unlike previous reports (Chang et al., 2002), we found significant changes in the expression of several pathways during the lag phase (*t* = 0 to *t* = 2 h). Metabolism pathways were strongly downregulated (**Figure 3B** bottom table and **Supplementary Table S2**) and in particular the most overrepresented of these KEGG pathways was “Microbial metabolism in diverse environments.” This pathway was previously found to play a key role in *Klebsiella pneumoniae* adaptation to cold or heat shocks (Tripathy et al., 2014). Among the 10 most downregulated genes, we found *astA* (-13.0 log_2_ fold change, squares in **Figure 4A**), *astB*, and *astC* in the AST pathway controlling arginine degradation; *gadA* and *gadB* controlling glutamate decarboxylase activity (De Biase et al., 1999); the biofilm regulator *bssR*; and the aldehyde dehydrogenase *aldB*. These genes were then all strongly induced during the exponential phase (between 8 and 10 log_2_ fold).

Among the upregulated pathways (**Figure 3B** top table and **Supplementary Table S2**), “Ribosome” was the most overrepresented indicating induction of the translation machinery. Furthermore, among the 10 most upregulated genes, we found *yghG* (7.9 log_2_ fold change, circles in **Figure 4A**) and *yghF* that were induced at *t* = 1 h and have previously been linked to type II secretion (Kim et al., 2017); *borD* encoding a prophage lipoprotein; *proV*, *proW*, and *proX* also induced at *t* = 1 h, encoding parts of an ABC transporter for the uptake of glycine, betaine, and proline; *iraM* induced at *t* = 1 h and encoding an anti-adapter protein that inhibits RpoS proteolysis; and *stpA* encoding a DNA-binding protein. *yghG*, *yghF*, *borD*, *iraM*, and *stpA* were then strongly downregulated (between -6 and -8 log_2_ fold) during exponential phase. Notably, gene-expression profiling during the lag phase was not reported in a previous transcriptomic study carried out on *E. coli* O157 (Bergholz et al., 2007).

During the exponential growth phase (*t* = 2 h to *t* = 5 h) there was an extensive reprogramming of gene-expression. The “Ribosome” pathway was the most overrepresented in the top 10% downregulated genes, indicating repression of the translation machinery at the transition between exponential and stationary phase (*t* = 5 h) in response to the depletion of nutrients in the culture conditions. This was reflected in the measured division rate (**Supplementary Figure S6**). Among the 10 most downregulated genes, we found *plaP* (-9.4 log_2_ fold change, squares in **Figure 4B**) encoding a putrescine importer required for the induction of pili-driven motility, in accordance with the reported low motility of exponentially growing *E. coli* (Amsler et al., 1993); *cspA* encoding a cold shock protein; *fhuF* encoding an iron reductase protein; *lpxT* encoding the lipid A 1-diphosphate synthase; *fecA* encoding an outer membrane receptor in the Fe^3+^ dicitrate transport system; and the above discussed *yghF*. *fhuF* and *plaP* were then upregulated by a factor of 6 and 2 log_2_ fold, respectively, during stationary phase.

Metabolism related pathways were upregulated with “Microbial metabolism in diverse environments” now being the most overrepresented KEGG pathway (**Figure 3C** top table). Among the 10 most upregulated genes, we found *gadA* (11.1 log_2_ fold change, circles in **Figure 4B**), *gadB*, *gadC*, and *gadE* that were induced at *t* = 4 h and whose upregulation at the transition between exponential and stationary phase have previously been reported (De Biase et al., 1999; Weber et al., 2005; Bergholz et al., 2007); *glcD* and *glcE* induced at *t* = 3.5 h, encoding a subunit of the glycolate oxidase; *narU* induced at *t* = 3 h, encoding a nitrate and nitrite inner membrane transporter; *aldB* already discussed above; and *tnaC* discussed below. *glcD*, *glcE*, and *tnaC* were then downregulated during the stationary phase. The *tnaC* gene is part of the *tnaCAB* operon that regulates tryptophan catabolism and is comprised of a 24 residue upstream peptide TnaC, the tryptophanase TnaA, and the low affinity tryptophan permease TnaB (Konan and Yanofsky, 1997). TnaA is responsible for the breakdown of tryptophan, which is utilized by *E. coli* as an energy source to produce pyruvate, ammonia, and indole (Luli and Strohl, 1990). Interestingly, as the sugars were depleted in the culture, we observed an increase in expression of the *tnaCAB* operon (**Supplementary Table S1**). This was in accordance with a previous proteomic study carried out on *E. coli* K12 BW25113 growing on minimal medium (Soufi et al., 2015), suggesting a correlation between tryptophan related gene and protein expression. Furthermore, Gaimster and Summers (2015) observed an increase in *tnaA* expression in a growing *E. coli* culture, correlating this to an increase in the concentration of extracellular indole (Gaimster et al., 2014). Finally, we also observed an upregulation of transport genes including *ompF* and *lamB* encoding two of the major *E. coli* outer membrane porins (**Supplementary Table S1**), which was not previously observed (Bergholz et al., 2007).

Metabolism related pathways were downregulated as the population entered stationary phase (*t* = 5 h to *t* = 7 h) with “Microbial metabolism in diverse environments” now being the most overrepresented KEGG pathway (**Figure 3D** bottom table). This coincided with the previously mentioned reduction in sugar availability (**Figure 1B**). Almost all of the genes in the phosphoenolpyruvate (PEP)-dependent phosphotransferase system (PTS) pathway, a major bacterial mechanism for the accumulation of carbohydrates (Shimizu, 2013), were downregulated as the bacteria moved from late-exponential to stationary phase (**Figure 3D**). The downregulation of the PTS pathway may cause reduced levels of glycolysis intermediates such as fructose 1,6-bisphosphate (FDP), which in *E. coli* results in the activation of *cra* and the subsequent transcriptional repression of *pfkA* and *pykF* (Shimizu, 2013). Both *pykF* and *pfkA* were downregulated as the culture transitioned from exponential to stationary phase (**Supplementary Figure S7**) resulting in the downregulation of the TCA cycle (**Figure 3D**). This is in agreement with a previous transcriptomic study carried out on *E. coli* O157, reporting a downregulation of the tricarboxylic acid (TCA) cycle pathway after *t* = 4.5 h compared to *t* = 3 h growth on minimal medium (Bergholz et al., 2007). Similarly, we found agreement between Bergholz’s data and ours on the downregulation of the sulfur metabolism pathway, sulfur being present in LB and instrumental for the biosynthesis of the amino acids cysteine and methionine (Sekowska et al., 2000).

Among the 10 most downregulated genes were *glcD* (-5.1 log_2_ fold change, squares in **Figure 4C**), *glcE*, *glcA*, and *glcB*; *ansB* encoding L-asparaginase 2; *fumB* encoding a fumarate hydratase; and *adiY* encoding a transcriptional regulator. *glcD*, *glcE*, *glcA*, and *glcB* were further downregulated during the lag phase. Finally, genes encoding transporters including *ompF* and *lamB*, were also downregulated as previously reported (Chang et al., 2002).

The “Ribosome” pathway was the most overrepresented in the top 10% upregulated genes. However, the mean expression of all the 48 genes in this pathway was downregulated by a factor of 2.8 log_2_ fold at *t* = 7 h compared to *t* = 2 h. Among the 10 most upregulated genes, we identified *fhuF* (6.1 log_2_ fold change, circles in **Figure 4C**); *astA*, *astC*, and *astE* in the AST pathway, that were induced at *t* = 6 h; *prpB* induced at *t* = 4 h, encoding the 2-methylisocitrate lyase; *bfd* encoding bacterioferritin-associated ferredoxin; *rmf* induced at *t* = 4 h, encoding a ribosome modulation factor; *ynfM* encoding an inner membrane transporter; *sodA* induced at *t* = 6 h, encoding a superoxide dismutase, previously associated with the emergence of metabolic heterogeneity during nutrient starvation (Şimşek and Kim, 2018); and *obgE* encoding the essential GTPase ObgE/CgtA. In the AST pathway, *rmf*, *ynfM*, and *prpB* were then strongly downregulated (between -5 and -10 log_2_ fold) during lag phase. Furthermore, a major regulator of the stress response in bacteria, particularly their entry into stationary phase, is the sigma factor *rpoS* controlling the expression of approximately 10% of genes in *E. coli* (Weber et al., 2005). Our data shows that *rpoS* expression increases rapidly as the culture enters stationary phase (**Supplementary Figure S8**), in accordance with previously reported data (2.4 and 2.8 log_2_ fold change, respectively, between *t* = 4 h and 5 h from inoculation) (Bergholz et al., 2007). Bergholz, et al. also reported that the most highly upregulated gene during the transition to stationary phase was *acs*, which encodes acetyl CoA synthetase, confirming the data reported in a separate study on *E. coli MG1655* (Baev et al., 2006). Similarly, between *t* = 4 h and *t* = 4.5 h from inoculation, we observe a 7.4 log_2_ fold change in the expression of *acs* and 4.7 log_2_ fold change of *aceB* expression, in accordance with Bergholz, et al. who reported a 5.4 log_2_ fold change for *aceB* during the same temporal window. These data suggest that at least some *E. coli* responses to changes in growth medium are conserved across evolutionary distance and are not specific to the growth medium employed. All together, our data on the reprogramming of the culture gene-expression during the transitions between the different growth phases (**Figures 3**, **4** and **Supplementary Tables S1**, **S2**) will be relevant for studying the responses of microbial communities to environmental changes.

### Growth Stage Dependent Persister Formation

We then studied the growth cycle dependence of phenotypic heterogeneity within the population by measuring persistence to antibiotics as a phenotypic proxy. We used three antibiotics with distinct modes of action: ampicillin, gentamicin, and ofloxacin. Specifically, ampicillin is a β-lactam that binds to the penicillin-binding proteins located inside the bacterial cell wall. It inhibits the last stage of bacterial cell wall synthesis leading to lysis mediated by autolytic enzymes. Gentamicin is an aminoglycoside that works by irreversibly binding to the 30S subunit of the bacterial ribosome, thereby interrupting protein synthesis. Ofloxacin is a second-generation fluoroquinolone that acts on DNA gyrase and topoisomerase IV, and thus altering the control of DNA supercoiling and inhibiting normal cell division (Aldred et al., 2014).

To investigate the growth-dependent heterogeneity of the response to each antibiotic, we performed two different treatments (see **Supplementary Figure S1**): three culture aliquots were injected with antibiotic and fresh LB (**Supplementary Figure S1F**), while three other aliquots were injected with antibiotic only (**Supplementary Figure S1G**). In both cases, the final antibiotic concentration was 25× the antibiotic MIC. For each time point and each antibiotic treatment, we then calculated the ratio between the measured number of persisters in the culture relative to the total number of bacteria in the culture (**Figure 3A**), defining this as the persister fraction. We finally normalized each persister fraction dataset to their maximum values.

**FIGURE 5:**
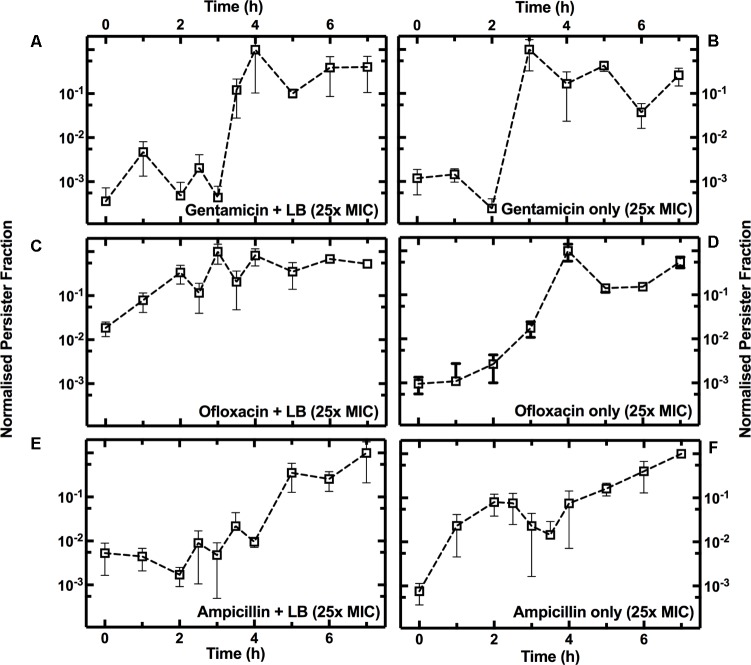
Growth phase dependence of the fraction of persisters to gentamicin, ofloxacin, or ampicillin. Temporal dependence of the normalized fraction of persisters to treatment either with gentamicin **(A)**, ofloxacin **(C)**, or ampicillin **(E)**, with the addition of fresh LB, or with antibiotics only [**(B,D,F)**, respectively]. At *t* = 0 an overnight *E. coli* culture was diluted 1:1000 in LB medium and the culture growth started. Each data set is normalized to its maximum persister fraction. Data and error bars are the mean and SEM of measurements obtained at least on biological and technical triplicates.

When gentamicin was added to the culture aliquots with fresh LB, the fraction of persisters showed a 3.4 log_10_ fold increase between *t* = 3 h and *t* = 4 h, before remaining relatively constant for the remainder of the growth cycle (**Figure 5A**). When only gentamicin was added to the culture aliquots the fraction of persisters showed a similar pattern, except for a shift of 1 h, with a 3.6 log_10_ fold increase between *t* = 2 h and *t* = 3 h (**Figure 5B**).

When ofloxacin was added to the culture aliquots with fresh LB, there was a small increase in the persister fraction during the lag phase (**Figure 5C**). However, the persister fraction showed a 2.6 log_10_ fold increase during the exponential phase (between *t* = 2 h and *t* = 4 h) when only ofloxacin was added to the culture aliquots (**Figure 5D**).

When ampicillin was added to the culture aliquots with fresh LB, we measured a 2 log_10_ fold increase in the persister fraction during the stationary phase (**Figure 5E**). When only ampicillin was added to the culture aliquots, we measured a 1.5 log_10_ fold increase in the fraction of persister cells during the lag phase (*t* = 1 h, **Figure 5F**).

Growth-dependent bacterial susceptibility has recently been reported (Greulich et al., 2015). Here, we demonstrate that as the composition of the medium in the culture environment changes, the microbial population becomes increasingly heterogeneous in response to the treatment to antibiotics with different modes of action.

Furthermore, antibiotic susceptibility and persister assays are often carried out by supplementing antibiotics with fresh LB medium (Wu et al., 2015; Orman and Brynildsen, 2016). We demonstrate that in the case of gentamicin, the addition of fresh LB medium does not substantially affect the dependence of persister fraction on growth phase. Indeed, it has recently been demonstrated that nutrient-rich environments do not increase susceptibility to antibiotics that irreversibly bind to the 30S subunit of the bacterial ribosome (Greulich et al., 2015). On the contrary, we observed that the formation of persisters to β-lactams and quinolones is strongly affected by the medium composition, suggesting that this should be carefully considered when screening for antibiotics against persister cells.

## Discussion

Within an isogenic population, there is inherent phenotypic heterogeneity which allows an adaptive response to an ever-changing extracellular environment (Balaban et al., 2004; Ryall et al., 2012; Nikolic et al., 2013; Kotte et al., 2014). For example, within a growing isogenic population of bacteria there are multiple growth phenotypes present, from exponentially growing to slow growing, or dormant bacteria (Ryall et al., 2012; Kotte et al., 2014). Persister cells are an example of a phenotype which differs from the majority of cells in a clonal population in terms of growth rate (Lewis, 2010; Maisonneuve et al., 2013), motility, gene expression, and cell size (Ryall et al., 2012). Furthermore, persister cells can be generated in response to a number of environmental conditions (Keren et al., 2004b; Vega et al., 2012; Bernier et al., 2013; Helaine et al., 2014), including nutrient limitation (Fung et al., 2010; Maisonneuve and Gerdes, 2014) and nutrient transitions (Amato et al., 2013) that also generate a variety of other bacterial responses (Lidstrom and Konopka, 2010).

Therefore, we utilized the persister phenotype as a proxy for changes in population-wide heterogeneity throughout the *E. coli* growth cycle where the environmental conditions are constantly changing. We observed homogeneity in response to all antibiotics during lag phase with very few persisters to any of the tested antibiotics. However, the different persister fractions observed in response to the different antibiotics further emphasize the phenotypic heterogeneity within the population during the exponential and stationary phases. In fact, population based heterogeneity allows rapid response to alterations in the nutritional environment; it is only when the environment becomes favorable to a given subpopulation that they are able to dominate the whole population-level response (Lidstrom and Konopka, 2010). Indeed, within the growth cycle we observed changes in both nutrient availability (**Figure 1**) and pH (**Supplementary Figure S4**).

These changes in the culture medium also influenced the population transcriptome during the same temporal windows where we measured notable increases in the fraction of persisters. We observed upregulation of carbon fixation pathways and tryptophan metabolism (**Figure 3C**), potentially as a result of an increase in the concentration of extracellular indole (Gaimster et al., 2014). Indole has also been linked to persister cell formation (Vega et al., 2012, 2013) and the induced expression of a variety of drug exporters (Meng and Bennett, 1992; Balaban et al., 2004). Moreover, *ompF* and *lamB*, encoding two of the major outer membrane porins that have been associated with drug uptake (Ziervogel and Roux, 2013; Lin et al., 2014; Cama et al., 2015) were also upregulated at the whole population level during exponential phase (**Supplementary Table S1**).

In comparison, during stationary phase we observed a clear downregulation of metabolism related pathways (**Figure 3D**). The downregulation of the TCA cycle as the population moves from exponential to stationary phase (**Figure 3D**) results in the excretion of acetate into the extracellular environment and its subsequent utilization (**Figure 1**; Luli and Strohl, 1990; Shimizu, 2013). Kotte et al. (2010) modeled population adaptation to different nutrients *in silico*, showing that as glucose levels reduce, cells are predicted to utilize their natively produced acetate. This ability to adapt to nutrient availability appears to be a result of metabolic flux at the single-cell level (Kotte et al., 2010; Kochanowski et al., 2013) and results in the diversification of growing and non-growing phenotypes, such as persisters (Kotte et al., 2014). Indeed, we measured an increase in persister fraction in response to all three antibiotics as the available sugars become limited (**Figure 1**). However, each type of antibiotic reveals different levels of heterogeneity suggesting that different biological pathways underlie persistence to different antibiotics.

We also found that the outcome of antibiotic treatment is strongly influenced by the composition of the medium containing the antibiotic. In fact, the addition of antibiotics and fresh LB alters the native culture environment and causes a reduction in the number of persisters (**Figure 5**), reducing phenotypic heterogeneity within the *E. coli* community.

One of the current limitations within our knowledge of persister bacteria is that their transcriptome has been examined only after treatment with antibiotics (Keren et al., 2004b) owing to the lack of biomarkers to isolate persisters from the majority of susceptible cells before antibiotic challenge. However, antibiotic treatment is known to alter the bacterial transcriptome (Lewis, 2010). Our current study identifies molecular pathways that are strongly regulated at the whole population level when the environment changes and, coincidentally, the fraction of persisters within the population increases. Some of the identified pathways such as tryptophan metabolism and TCA cycle (Vega et al., 2012; Kotte et al., 2014), have indeed previously been associated with persisters. However, it is noteworthy that our approach measures the mean transcriptomic response of the whole population. Therefore, our measurements do not allow us to determine whether the pathways that we have identified are also strongly regulated in the minority of persister cells. Indeed, the differential response of persisters could be masked by that of the majority of susceptible cells. Considering that cell-to-cell variation increases with increasing mean gene-expression (Silander et al., 2012), these comprehensive data sets provide well defined culturing time points, medium compositions, and putative pathways that could be investigated with single-cell approaches (Henry and Brynildsen, 2016; Bamford et al., 2017) to determine molecular pathways that are differentially regulated in persisters compared to the majority of susceptible cells.

Our approach could easily be extended to investigate the dynamics of phenotypic heterogeneity in different microbial communities such as bacterial biofilms (Domka et al., 2007), natural yeast and fungal populations (LaFleur et al., 2010; Holland et al., 2014), or cancer cells (Hangauer et al., 2017) responding to a variety of environmental cues.

## Author Contributions

SP developed the project and designed the research. AS, AK, RB, CS, SF, and AK-S performed the experiments. PO, KM, and KP carried out sequencing. All authors analyzed and discussed the data. AS and SP wrote the paper.

## Conflict of Interest Statement

The authors declare that the research was conducted in the absence of any commercial or financial relationships that could be construed as a potential conflict of interest.
