# Supplementary material for: The Culture Environment Influences Both Gene Regulation and Phenotypic Heterogeneity in Escherichia coli
Source: Front Microbiol. 2018 Aug 15;9:1739. doi: 10.3389/fmicb.2018.01739 (PMC6104134; doi:10.3389/fmicb.2018.01739)
Supplement: Supplementary file 3 [file Image_1.pdf]

![frontiers]

Supplementary Material

The culture environment influences both gene regulation and phenotypic heterogeneity in *Escherichia coli*

Ashley Smith[1,2], Agnieszka Kaczmar[1,2], Rosemary A. Bamford[1,2], Christopher Smith[2], Simona Frustaci[1], Andrea Kovacs-Simon[2], Paul O'Neill[2], Karen Moore[2], Konrad Paszkiewicz[2], Richard W. Titball[2], Stefano Pagliara[1,2]*

[1]Living Systems Institute, University of Exeter, Exeter, UK
[2]Biosciences, University of Exeter, Exeter, UK

*** Correspondence:**
Stefano Pagliara
s.pagliara@exeter.ac.uk

1 Supplementary Figures and Tables

1.1 Supplementary Tables

Table S1. A comprehensive data set of gene expression profiling at the different phases of growth. Columns A-D report each gene b, JWID, and ID number and name, respectively. DESeq2 was used to normalize the raw transcript reads using the median of ratios method and correcting for library size. Columns E and F report the mean, and standard error of the mean, calculated by averaging measurements of normalized transcript reads obtained from three t=17 h culture aliquots. Columns G-Z report the mean, and standard error of the mean, of the $\log_2$ fold change in normalized transcript reads on the samples at t=0, 1, 2, 3, 3.5, 4, 4.5, 5, 6, and 7h post dilution, respectively, relative to the normalized transcript reads of the t=17h sample.

Table S2. Overrepresented pathways during each growth phase. The table reports the top 20 overrepresented KEGG pathways. The top 10% of up or down differentially expressed genes were selected and goseq used to identify the overrepresented KEGG pathways for each phase of growth. Column A reports if these pathways were up or down regulated. Column B reports the name of the pathway. Column C gives the calculated p value. Column D shows the number of genes that were differentially expressed (number of genes that were in the top 10% of up or down regulated genes, respectively). Column E reports the total number of genes in the pathway.

1.2 Supplementary Figures

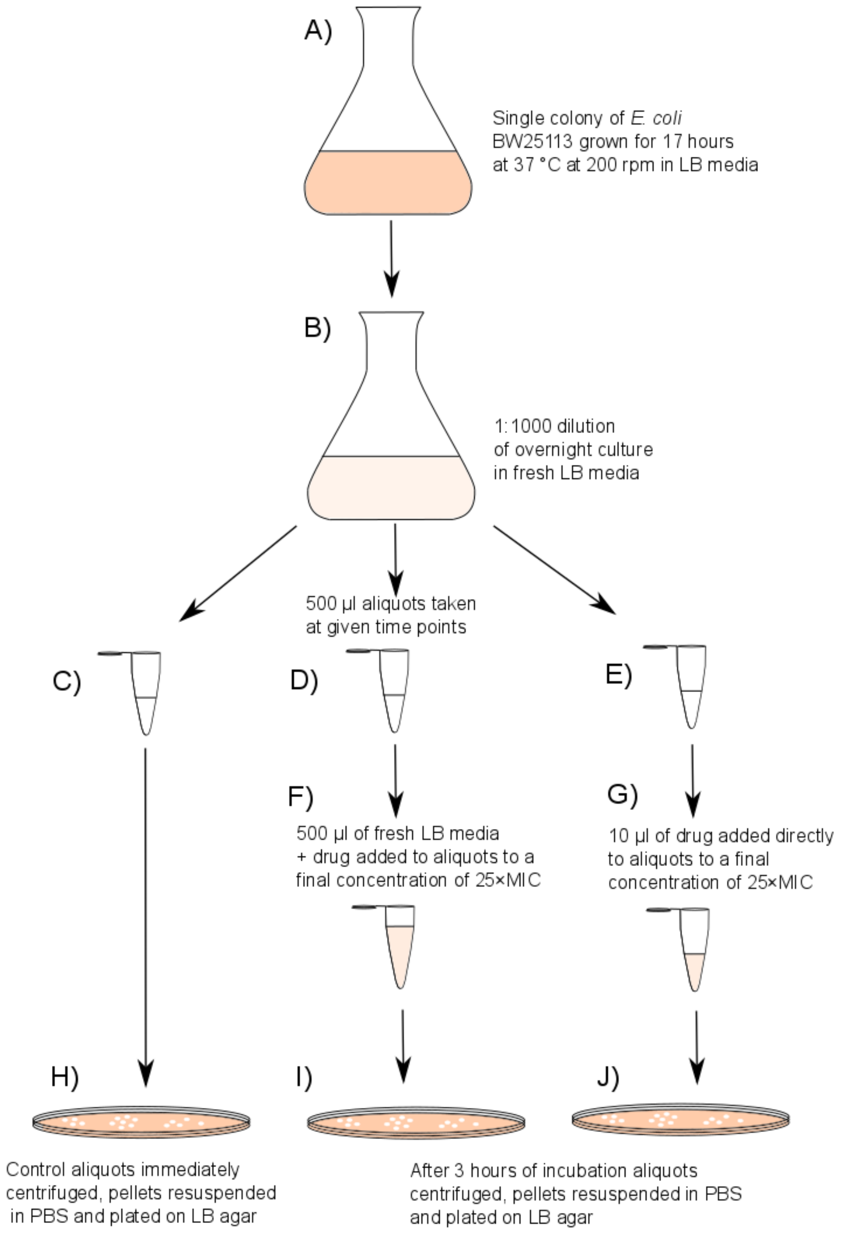

Figure S1. Schematic illustrating the persister assay. A single colony of *E. coli* was grown at 37 °C and 200 rpm in 200ml LB for 17 hours (A). We defined t=0 the time at which this culture was diluted 1:1000 in LB and growth at 37 °C and 200 rpm was restarted (B). Nine aliquots were taken from the growing culture hourly (C-E). Three aliquots were centrifuged immediately (13,000 *g*, 5 minutes) (C), serially diluted and plated on LB agar (H). The other six were treated for 3 hours, three supplemented with 500 µl of fresh LB (F) and three directly to the aliquot (G). In both cases the final concentration was 25×MIC. The treated aliquots were incubated for 3 hours, centrifuged, re-suspended in PBS and plated on LB agar(I-J).

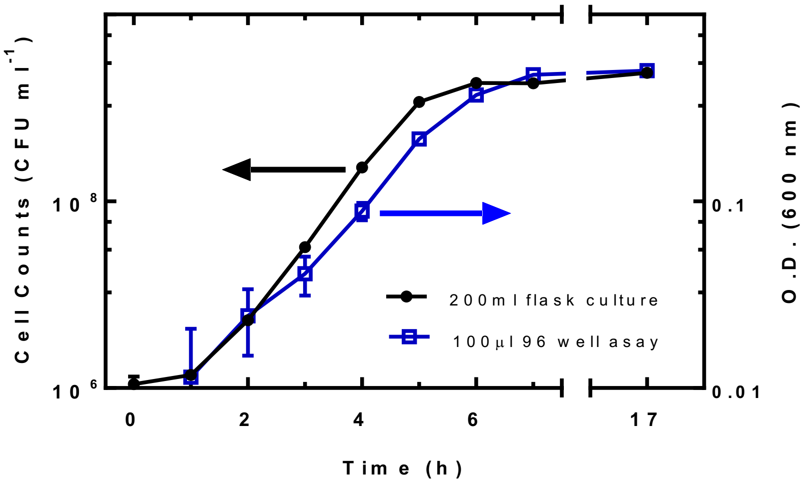

Figure S2. Comparison of *E. coli* growth in a conical flask and in a 96 well plate. Three overnight cultures of *E. coli* were diluted 1:1000 in fresh LB. Three 200 mL aliquots of these solutions were placed in three glass conical flasks whereas eighty-one 100 µl aliquots were added to individual wells of a 96 well plate (3 technical replicate in biological triplicate for each of the 9 time points investigated). The remaining wells were filled with fresh LB for blank measurements. Growth was measured hourly via CFU counts on LB agar plated with technical triplicate taken hourly from each flask (left axis) and via O.D. 600 measurements of 9 selected wells on a plate reader (right axis). All data represent mean and standard error of the mean of biological and technical triplicates. Some error bars are small compared to the corresponding mean values and are hidden behind the data.

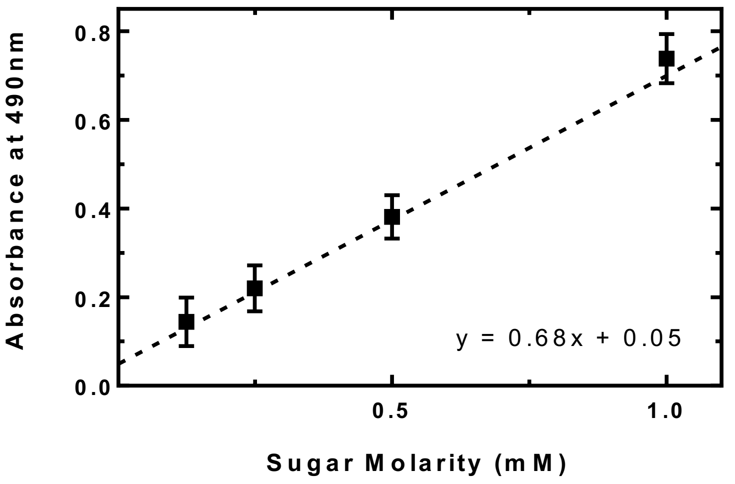

Figure S3. Standard curve for glucose detection using Benedicts reagent. The absorbance at 490 nm was measured for known glucose concentrations of 125, 250, 500 and 1000 µM, respectively. The background was determined as the average of a triplicate measure of milliQ water. Triplicate readings were taken for each concentration, the background subtracted and the mean and SEM plotted. A linear regression was fitted to these points and the resulting function used to extrapolate sugar concentrations through the growth cycle of *E. coli.*

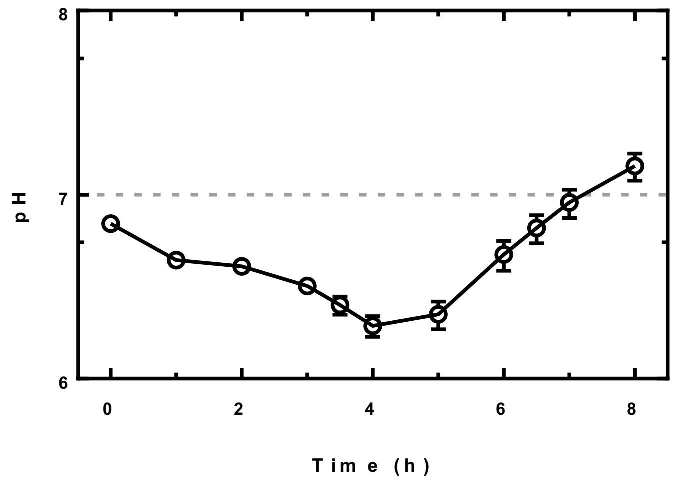

Figure S4. The growing culture shapes the extracellular environment pH. An overnight culture of *E. coli* was diluted 1:1000 in fresh LB and the pH of the culture measured through the resulting growth cycle. The pH decreases during lag and early exponential phase reaching a minimum of 6.2, then increases during late-exponential phase and up to 7 after 7h of growth. Data and error bars are the mean and SEM of at least 3 biological replicates.

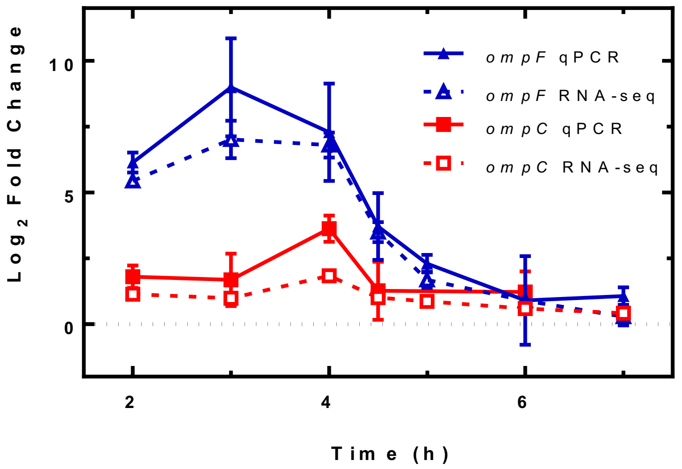

Figure S5. qPCR measurements confirm reliability of RNA-seq. In order to confirm the reliability of the RNA-seq data we performed quantitative PCR (qPCR) on the same samples for selected genes. The open symbols and dashed lines represent the transcriptomic results, whereas the filled symbols and lines represent those values obtained from qPCR. The results clearly indicate a similar pattern in the expression profiles for both methods.

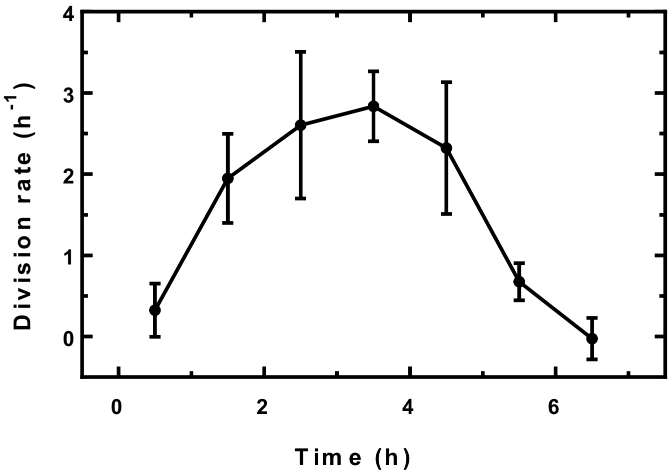

Figure S6. The bacterial division rate throughout the different stages of growth. An overnight culture of *E. coli* BW25113 was diluted 1:1000 in fresh LB. We measure division rate as the log_2 fold change between the bacterial CFU at consecutive hourly intervals throughout the bacterial growth cycle reported in Fig. 2A. Means and SEM are calculated from at least 3 biological and technical replicates.

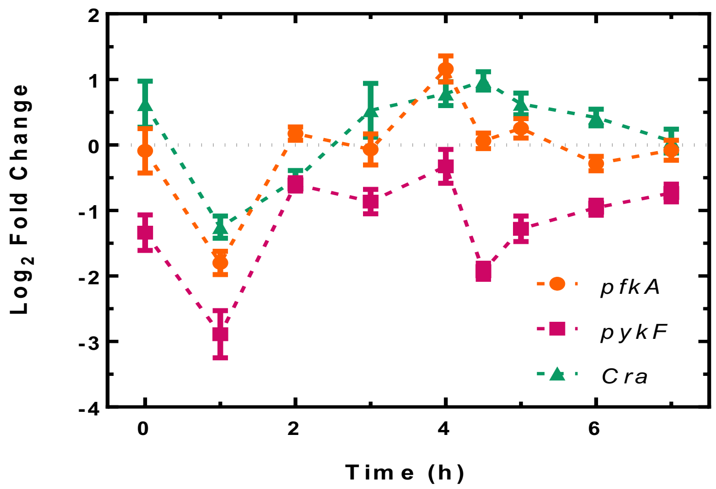

Figure S7. Regulation of glycolysis related genes. Carbon starvation results in increased expression of the transcription factor *Cra* (green triangles) which represses glycolysis enzymes *pfkA* (orange circles) and *pykF* (purple squares). During carbon starvation in *E. coli* levels of the glycolysis intermediate phosphoenol pyruvate (PEP) increases and inhibits the enzyme *pfkA*, which catalyzes an earlier step of glycolysis where GF6P is converted to FDP. The resulting increase in FDP activates the transcription factor *cra* which subsequently represses both *pykF* and *pfkA*.

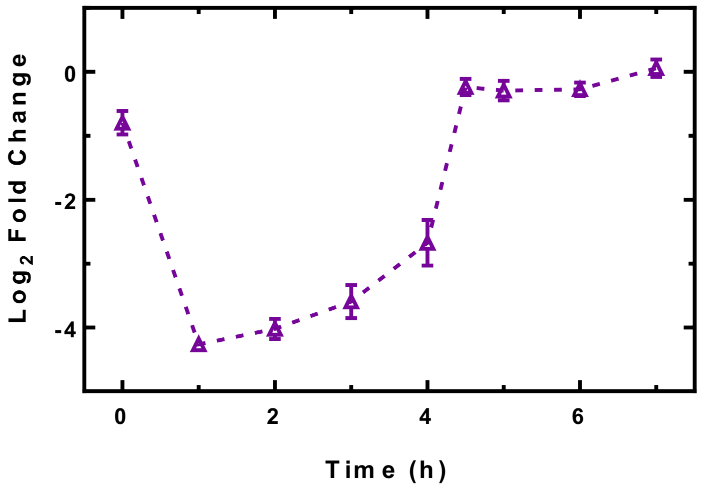

Figure S8. The regulation of the sigma factor *rpoS* through the growth cycle. *rpoS* is a sigma factor that is heavily associated with adaptation to stress and the onset of stationary phase. The graph above shows how upon dilution into fresh, nutrient rich, LB media, *rpoS* is downregulated. As the growth cycle progresses, it is gradually upregulated before a sharper rise is observed at the onset of stationary phase, where it then plateaus.
